# CT-Based Liver Segmentation for Liver Surgery: A Hybrid Approach Based on 3D U-Net–ELM Model

**DOI:** 10.3390/biomedicines14061298

**Published:** 2026-06-07

**Authors:** Zeki Ogut, Eser Sert, Ertugrul Kaya, Muhammed Yildirim

**Affiliations:** 1 Department of Surgery, Elazig Fethi Sekin City Hospital, Elazig 23300, Türkiye; drzeki44@gmail.com; 2 Department of Computer Engineering, Malatya Turgut Ozal University, Malatya 44210, Türkiye; eser.sert@ozal.edu.tr; 3 Computer Programming Program, Department of Computer Technologies, Kahramanmaraş Sutcu Imam University, Kahramanmaraş 46050, Türkiye; ertugrulkaya@ksu.edu.tr; 4 Department of Artificial Intelligence and Data Engineering, Firat University, Elazig 23119, Türkiye

**Keywords:** 3D U-Net, extreme learning machine (ELM), hybrid deep learning, liver segmentation, computed tomography (CT), medical image segmentation

## Abstract

**Background:** Accurate liver segmentation from abdominal computed tomography (CT) images is an important task for surgical planning, volumetric analysis, and tumor assessment. Although recent deep learning-based three-dimensional segmentation approaches provide high segmentation performance, these models generally require high computational resources and long training times. **Methods:** In this study, a hybrid liver segmentation framework combining the 3D U-Net architecture with the extreme learning machine (ELM) method was proposed. In the proposed approach, deep volumetric feature maps extracted from the bottleneck layer of the trained 3D U-Net were used as input to an ELM-based classifier for final segmentation refinement. All experiments were performed on the Task03_Liver_rs dataset, which is a rescaled version of the Medical Segmentation Decathlon liver dataset. To provide a more reliable evaluation, fivefold cross-validation experiments were conducted using the same preprocessing pipeline, training protocol, and hyperparameter settings for all comparison models. In addition to overlap-based metrics, boundary-based and clinically relevant metrics including HD95, ASD, surface Dice, and volumetric error were also evaluated. **Results:** Experimental results demonstrated that the proposed 3D U-Net–ELM framework achieved competitive and stable segmentation performance compared with nnU-Net, standard 3D U-Net, SwinUNet, and SwinUNet–ELM models. The proposed model achieved a mean Dice score of 0.9399 ± 0.0210 and an IoU score of 0.8874 ± 0.0358 under fivefold cross-validation. Furthermore, the proposed approach produced lower HD95 and ASD values together with higher surface Dice scores, indicating improved boundary consistency and volumetric segmentation quality. In addition, the hybrid ELM-based structure provided advantages in computational efficiency and training cost. **Conclusions:** The obtained findings indicate that the proposed 3D U-Net–ELM framework provides a balanced and computationally efficient alternative for volumetric liver segmentation. Nevertheless, the absence of independent multicenter external validation remains an important limitation of the study. Future studies will focus on evaluating the proposed framework using larger and more diverse multicenter datasets to further investigate its clinical applicability and generalizability.

## 1. Introduction

Liver segmentation is a crucial area of study in hepatobiliary surgery for patient management. Accurate and reliable determination of liver boundaries is vital for preoperative liver volume analysis, calculating future liver remnant (FLR) volume, evaluating resections, planning graft volume for transplantation, and quantitatively analyzing tumor burden [1,2]. Even small deviations in segmentation measurements can affect volume calculations. Furthermore, these deviations can lead to changes in surgical approach, differing resection decisions, and misjudgment of the risk of postoperative liver failure [3,4]. Similarly, the accuracy of volumetric analyses performed during recipient, and especially living donor evaluations, in liver transplantation is significantly affected by the quality of segmentation [4,5]. Therefore, segmentation quality is considered a critical factor for the accuracy of surgical strategy and patient safety [1,6].

In clinical practice, liver segmentation is considered a fundamental component in planning treatment and evaluating treatment response in liver cancers [1,7]. Accurate determination of liver volume and tumor burden is crucial for predicting surgical risk, determining transplantation requirements, calculating radiotherapy doses, and planning regional treatment strategies [8,9]. In particular, the reliable monitoring of the targeted downstaging process after transarterial chemoembolization, transarterial radioembolization, and other regional treatments applied in the treatment of hepatocellular carcinoma (HCC) is closely related to the accuracy of segmentation-based measurements [10]. Problems in liver segmentation can lead to errors in the clinician’s calculation of tumor size and liver volume, resulting in incorrect treatment planning. Therefore, segmentation accuracy is critically important for patient safety and the reliability of treatment decisions [1,4].

With the advancement of technology, imaging technologies have also experienced breathtaking developments in recent years, and as a result, they have been frequently used in recent years for the diagnosis of diseases, treatment planning, and guiding surgical interventions [11,12]. Three-dimensional imaging techniques such as computed tomography (CT) and magnetic resonance imaging (MRI) provide high-resolution visualization of the anatomical structure of the liver. However, automatically, accurately, and reliably extracting organ boundaries from these images is a challenging process. Anatomical differences between individuals, morphological variability between lesions and tumors, tissue heterogeneity, intense boundary relationships with neighboring organs, and low contrast or noise levels in the images make liver segmentation difficult. For these reasons, even manual segmentation performed by clinicians can result in significant inconsistencies. Therefore, accurately segmenting the complex boundary structure and low-contrast regions of the liver using automated methods is a challenging process. Traditional methods, such as thresholding, region enlargement, or active contour-based models that exist in the literature, are unfortunately often insufficient for modeling such complex variations and consequently require intensive manual intervention and do not demonstrate successful generalization ability [13].

In recent years, convolutional neural networks (CNNs) in particular have led to significant advances in the field of medical image segmentation, and segmentation performances have increased considerably [14]. In this context, 3D CNN-based models enable the preservation of spatial context and more comprehensive feature extraction by directly addressing the volumetric structure of medical images. Three-dimensional convolution operations are particularly suitable for modeling the three-dimensional structure of complex organs such as the liver. However, the high computational cost, memory usage, and need for large datasets of 3D networks can limit the practical application of these methods in clinical settings [15]. Therefore, the development of models optimized in terms of both accuracy and computational efficiency is an important ongoing research topic in the field of medical image segmentation.

In this context, the UNet architecture proposed by Ronneberger et al. [16] enables precise segmentation results by combining features at different levels thanks to its encoder–decoder structure. Later, a version of this architecture adapted to volumetric data, 3D UNet [15], was developed by Çiçek et al., and high-accuracy segmentation results were obtained on 3D CT and MRI data. However, training 3D UNet models requires intensive computational power and long training times. In addition, factors such as data quality, the limited number of labeled data, and noise can affect model performance. Therefore, hybrid approaches that improve the performance of deep feature extractors like 3D UNet and at the same time reduce the computational load are increasingly being investigated in the literature.

Extreme learning machine (ELM) is mostly used in the literature as a complementary approach to deep learning. This model is a single-layer feedforward neural network (SLFN) model developed by Huang et al. in 2004 [17]. The basic principle of the model is based on the random assignment of input weights and the analytical calculation of only the output weights. In this way, ELM can offer much shorter training times compared to traditional backpropagation-based methods [18]. Therefore, ELM is used together with deep learning models, especially to increase computational efficiency in the classification phase after feature extraction.

In this study, a hybrid method combining deep learning-based feature extraction with analytical learning is proposed to address the liver segmentation problem in three-dimensional medical images. Experimental studies were carried out on the Task03_Liver_rs [19] dataset using a fivefold cross-validation strategy to comprehensively evaluate the performance, stability, and computational efficiency of the proposed approach. In the proposed structure, the 3D U-Net architecture is trained to extract high-level spatial and contextual features, and the resulting bottleneck representations are evaluated using an ELM-based decision mechanism rather than a classical decoder to generate voxel-level predictions. This approach offers an alternative framework that separates feature extraction from decision-making processes, unlike traditional end-to-end segmentation architectures. Therefore, the proposed structure should be considered not as a conventional end-to-end encoder–decoder segmentation network, but rather as a hybrid framework that evaluates learned volumetric representations using an alternative analytical learning strategy. In addition, the proposed framework was evaluated using both overlap-based and boundary-based metrics in order to provide a more comprehensive analysis of segmentation quality. Considering the high computational cost and overfitting tendency of backpropagation-based optimization, especially in the deeper layers of volumetric networks, the proposed framework aims to provide a balanced structure in terms of both segmentation performance and computational efficiency through the analytical solution mechanism offered by ELM. The main contributions of this study can be summarized as follows:

- A hybrid segmentation framework is proposed that combines the powerful feature-extraction capability of 3D U-Net with the fast, analytical learning approach of ELM. This structure, unlike classical encoder–decoder architectures, makes the decision mechanism partially independent of backpropagation.
- It has been shown that processing representations obtained from the bottleneck layer using ELM can provide an advantage in terms of computational cost and deliver competitive results in terms of segmentation performance.
- The proposed method was evaluated using fivefold cross-validation and compared with current CNN- and transformer-based segmentation models using both overlap-based and boundary-based evaluation metrics.
- The proposed framework was evaluated not only in terms of segmentation accuracy but also with respect to computational efficiency, training cost, and boundary consistency, demonstrating a competitive and resource-efficient alternative for volumetric liver segmentation.
- Although the proposed framework demonstrated stable results under fivefold cross-validation experiments, further multicenter external validation studies are still required to comprehensively evaluate the generalizability and clinical applicability of the proposed method.

In recent years, significant progress has been made in the field of medical image segmentation with the widespread adoption of deep learning methods. In particular, architectures based on convolutional neural networks offer effective solutions for the automatic analysis of complex anatomical structures in medical images. One of the most effective architectures in this field is the U-Net model proposed by Ronneberger, Fischer, and Brox. Thanks to its encoder–decoder structure and skip connections, U-Net can effectively combine high-resolution spatial information with contextual features learned in deep layers, thus obtaining precise segmentation results [16].

The success of the U-Net architecture was later improved by adapting it to volumetric medical data. 3D U-Net, proposed by Çiçek et al., can model contextual relationships between adjacent sections by replacing all two-dimensional operations with their three-dimensional counterparts, and can obtain highly accurate segmentation results even with a limited number of labeled data [15]. V-Net, an important architecture based on three-dimensional convolutions, was proposed by Milletari et al., and offers an effective solution in class imbalances with its structure, which optimizes Dice performance [20]. This architecture has provided significant improvements in accuracy and generalization performance, especially by performing end-to-end volumetric segmentation in prostate MR images. For these reasons, 3D U-Net and V-Net are preferred and used in comparison in many studies.

Over time, U-Net-based architectures have been diversified with various structural improvements. Models such as Residual U-Net, Dense U-Net, and Attention U-Net aim to improve performance by adding different block structures and attention mechanisms. For example, Attention U-Net uses attention gates to ensure that the model focuses only on relevant anatomical regions, achieving significant improvements in the segmentation of small or irregular structures [21]. In addition, some studies have achieved more successful results in complex anatomical regions by developing two-stage architectures that perform coarse segmentation and fine detail segmentation in separate stages.

In recent years, Transformer-based architectures have become a significant research direction in the field of medical image segmentation. The TransUNet (2021) model developed by Chen et al. can capture long-range dependencies by processing feature maps obtained with a convolution-based encoder with Vision Transformer and can generate detailed segmentation masks with a U-Net-like decoder [22]. Similarly, the Swin-UNet (2021) model proposed by Cao et al. can effectively model global contextual information thanks to Swin Transformer blocks with a shifted window mechanism [23].

One of the important Transformer-based models developed for three-dimensional volumetric segmentation is the SwinUNetR architecture. Hatamizadeh et al. (2021) performed long-range context modeling on 3D data using a Swin Transformer-based encoder and ensured the preservation of fine details thanks to U-Net-style skip connections. This model achieved particularly high-level results in the BraTS 2021 brain tumor dataset. SwinUNetR-V2 developed later, and the model features an advanced architecture with convolutional components and has demonstrated high performance on large benchmark datasets such as Medical Segmentation Decathlon (MSD) [24].

To evaluate the performance of these architectures, datasets such as BraTS (brain tumors), LiTS (liver tumors), KiTS (kidney tumors), and MSD (multi-organ segmentation) are commonly used [25]. For example, the nnU-Net study (Isensee et al., 2021) achieved first place in many segmentation tasks by automatically optimizing data preprocessing and postprocessing steps with hyperparameters without making major changes to the architecture [26]. This result shows that a well-structured 3D U-Net derivative can still offer quite strong performance.

Deep learning-based segmentation methods are used in clinical applications in many different fields such as brain tumors, liver and pancreatic tumors, prostate, lung nodules and lymph node segmentation [20,25]. These methods offer significant advantages in terms of reducing the workload of radiologists, supporting treatment planning and improving early diagnosis processes. However, issues such as model reliability, generalization ability between different imaging devices and protocols, and model explainability are still important research topics in clinical applications.

In general, the medical 3D segmentation literature is developing along two main approaches. The first is U-Net-based and optimized derivatives (3D U-Net, V-Net, Attention/Residual/Dense U-Net, nnU-Net, etc.), and the second is Transformer-based next-generation architectures (TransUNet, Swin-UNet, SwinUNetR, etc.). The current research trend is to develop architectures that combine these two approaches in hybrid structures and can use both powerful feature extraction and global context modeling together. SegFormer3D (Perera et al., 2024), developed in this direction, offers a lightweight Transformer architecture that can achieve high accuracy while reducing computational cost thanks to its multi-scale attention mechanism and MLP-based decoder structure [27].

Similarly, the adaptive attention and multiscale feature fusion mechanisms proposed by Xiang et al. (2025) resulted in a significant performance increase in spine 3D segmentation compared to classical 3D CNN, 3D U-Net, and 3D U-Net + Transformer approaches [28]. This result demonstrates that similar multiscale and attention-based strategies can also be effective in complex volumetric tasks such as liver segmentation. Furthermore, the HResFormer model proposed by Ren and Li (2025) achieved superior results in 3D volumetric segmentation tasks compared to previous models thanks to the HLGM module combining 2D Transformer and 3D Transformer constructs and the residual learning strategy [29].

## 2. Materials and Methods

### 2.1. Dataset Description

The Task03_Liver_rs [19] dataset used in this study is a pre-processed version of the original MSD Task03_Liver dataset. In preparing this dataset, some examples with obvious image artifacts or labeling errors were removed. This data cleaning process was not carried out within the scope of this study and should be considered as a structural feature of the dataset used.

The original dataset contains a total of 131 labeled training volumes and 70 unlabeled test volumes. In this study, to improve data quality, some samples containing artifacts or masking errors were removed from the dataset, and a total of 123 labeled 3D CT volumes were used. All images in the dataset are provided in NIfTI format (.nii or .nii.gz). Each volume is used with its corresponding segmentation mask, where the liver region is labeled at the voxel level. Table 1 compares the original dataset with the version used.

To evaluate the learning and generalization capability of the proposed model in a more robust manner, fivefold cross-validation was employed throughout all experiments. In each fold, the dataset was divided at the patient level into training, validation, and testing subsets while preserving case independence between folds. The same fold configurations and preprocessing procedures were consistently applied to all compared models to ensure a fair and reproducible evaluation protocol. Since the Task03_Liver_rs dataset represents a downsampled version of the original MSD liver dataset, direct comparison with studies using the original high-resolution dataset should be interpreted carefully. Nevertheless, all evaluated models, including the nnU-Net baseline, were trained and tested under identical preprocessing, training, and evaluation settings to ensure a fair internal comparison.

### 2.2. Proposed Hybrid Segmentation Framework

**Figure 1:**
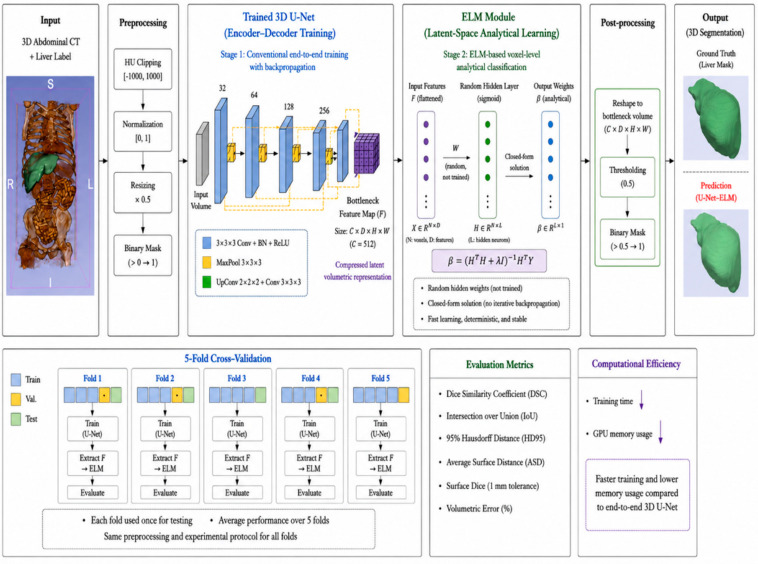
Flowchart of the proposed approach.

The method proposed in this study offers a hybrid structure that separates the decision-making process from deep feature extraction, unlike classical end-to-end segmentation architectures. In this context, the 3D U-Net architecture is primarily used as a deep volumetric feature extractor, while the final analytical decision mechanism is implemented using an ELM-based structure. Therefore, the proposed model should be considered a hybrid model in which the learned representation space is evaluated in an alternative way, rather than using traditional encoder–decoder-based segmentation approaches. This approach, unlike classical end-to-end learning structures, employs a two-stage learning strategy that separates feature extraction and decision-making. In the first stage, the classical encoder–decoder structure of the 3D U-Net architecture was trained in a conventional end-to-end manner using backpropagation in order to learn volumetric anatomical feature representations from CT images. After completion of the segmentation training process, bottleneck-level latent feature maps obtained from the encoder pathway were extracted and rearranged into voxel-wise feature vectors. In the second stage, these learned bottleneck representations were used as input to the ELM module, where voxel-level analytical classification was performed using the closed-form learning mechanism of ELM. Thanks to this separation, the representational power of the deep network is preserved, while the learning mechanism used in the decision layer can be optimized independently. Especially given the high computational cost, hyperparameter sensitivity, and tendency toward overlearning of backpropagation-based optimization in the last layers of deep net-works, ELM’s analytical solution offers a faster, deterministic, and more stable alternative at this stage. The fundamental difference of the proposed method is not only the sequential use of two different models, but also the re-evaluation of the high-dimensional, discriminative feature space obtained in the bottleneck layer using a non-gradient-based analytical learning mechanism. This aims to improve the model’s generalization, especially in limited-data scenarios, and make the training process more stable. Furthermore, thanks to the closed-form solution of ELM, the training time is significantly reduced, and the model’s computational efficiency is increased. The general flowchart of the proposed approach is shown in Figure 1. The method consists of the following steps: preparing the input data, deep feature extraction, obtaining bottleneck representations, generating feature vectors, performing an ELM-based learning process, and generating the final segmentation mask.

#### 2.2.1. Data Preprocessing

Before model training, several preprocessing steps were applied to the dataset in order to improve data consistency and stabilize the learning process. Medical image data may exhibit significant differences in density distribution, spatial resolution, and anatomical scale depending on imaging devices, acquisition protocols, and patient-specific variations. These variations can negatively affect the consistency of the data presented to deep learning models and may lead to unstable optimization behavior during training. Therefore, a standardized preprocessing pipeline consisting of data loading, resizing, density clipping, normalization, and mask configuration stages was applied within the scope of the proposed framework.

In the first stage, all CT image volumes and their corresponding segmentation masks were loaded into the system in NIfTI format (.nii/.nii.gz). To prevent potential mismatches between the image and label data, all image–mask pairs were verified using file-name consistency checks. Since three-dimensional medical images generally contain high-resolution volumetric data, direct training on the original volumes requires high GPU memory usage and significantly increases the computational cost and training time. Therefore, all CT volumes and corresponding segmentation masks were resized to approximately half resolution using a resize factor of 0.5. During this process, appropriate interpolation methods were used to preserve volumetric intensity continuity in CT images, whereas nearest-neighbor interpolation was applied to segmentation masks in order to preserve label integrity.

In computed tomography (CT) imaging, voxel intensity values are represented in Hounsfield units (HU), and these values can vary over a very wide range. Extremely high or low HU values may introduce instability and noise during model training. Therefore, voxel intensities were clipped to the range of [−1000, 1000] HU according to Equation (1):

(1) $$I_{clip}=\min\left(\max\left(I,\;-1000\right),\;1000\right)$$

where I represents the original voxel intensity value and I_clip denotes the clipped voxel intensity. After the clipping process, all CT volumes were normalized to a common density scale in order to improve numerical stability during optimization. In this study, voxel intensities were scaled to the range of [0, 1] using the normalization procedure defined in Equation (2):

(2) $$I_{norm}=\frac{\left(I_{clip}+\;1000\right)}{2000}$$

where I_norm represents the normalized voxel intensity value.

In the final stage of the preprocessing pipeline, segmentation masks were converted into binary format because the present study focused solely on liver segmentation. Accordingly, all voxel labels greater than zero were assigned the value 1 to represent liver tissue, while background voxels were assigned the value 0. The binary mask generation process can be expressed as follows:

(3) $$M_{bin\left(x\right)}=\;\left\{1,\;if\;M\left(x\right)>\;0\;;\;0,\;if\;M\left(x\right)=\;0\right\}$$

where M(x) represents the original segmentation label value at voxel position x, and M_bin(x) denotes the corresponding binary liver mask used during model training. As a result of these preprocessing stages, all CT volumes and corresponding segmentation masks were transformed into a standardized and computationally efficient representation suitable for the proposed hybrid segmentation framework.

#### 2.2.2. Preparation of Input Volumes

In the first stage of the proposed framework, the preprocessed CT volumes obtained from the preprocessing pipeline described in Section 3.2 were fed into the 3D U-Net architecture as volumetric input data. Each CT volume can be represented as a three-dimensional tensor, as expressed in Equation (4):

(4) $$X\;\in \;R^{H\;\times \;W\;\times \;D}$$

where H, W, and D denote the height, width, and depth dimensions of the volumetric CT data, respectively. Each voxel value corresponds to the normalized tissue density information at the associated spatial location. The resulting volumetric representations were then forwarded to the encoder pathway of the proposed 3D U-Net architecture for deep feature extraction.

#### 2.2.3. Deep Feature Extraction Using Trained 3D U-Net

In the third stage of the proposed framework, the preprocessed volumetric CT data are first trained using the classical encoder–decoder structure of the 3D U-Net architecture in order to learn hierarchical volumetric anatomical representations. The 3D U-Net architecture is a deep learning model that has demonstrated successful segmentation performance in volumetric medical imaging applications. The architecture consists of two main components: an encoder pathway and a decoder pathway. During the training stage, the encoder progressively extracts multi-level volumetric feature representations from the input data, while the decoder reconstructs segmentation-related spatial information through successive upsampling operations. In this way, the network learns both local anatomical structures and broader contextual volumetric information in an end-to-end manner.

Within the encoder pathway, volumetric feature extraction is performed using successive three-dimensional convolution operations. The feature extraction process in a convolution layer can be mathematically expressed as follows in Equation (5) [15]:

F^l^ = σ(W^l^ * F^(l−1)^ + b^l^) (5)

where F^(l−1)^ represents the feature map from the previous layer, W^l^ denotes the convolution kernel, b^l^ represents the bias term, * indicates the convolution operation, and σ(.) denotes the activation function defined in Equation (6). In this study, the rectified linear unit (ReLU) activation function was employed:

σ(x) = max(0,x)(6)

In the encoder architecture, a max-pooling operation was applied after each convolution block in order to progressively reduce spatial dimensions and increase the receptive field. The max-pooling operation can be expressed as follows:

Fpool(i,j,k) = max(u,v,w) ∈ Ω F(u,v,w)(7)

where Ω represents the pooling window used during the max-pooling operation. This hierarchical structure enables the model to learn both low-level anatomical patterns and high-level contextual volumetric representations. In the proposed architecture, the number of feature channels was progressively increased throughout the encoder pathway. The first encoder block employed 32 feature maps, followed by 64, 128, and 256 feature maps in subsequent levels.

After completion of the conventional encoder–decoder training process, bottleneck-level latent feature maps learned by the encoder pathway were extracted and used as compact volumetric representations for the subsequent ELM-based analytical learning stage. In this framework, the decoder pathway primarily served as a representation-learning component during the end-to-end training stage of the 3D U-Net architecture. Final voxel-level segmentation predictions were generated by the ELM module operating on bottleneck latent representations rather than directly using the original decoder output.

In the proposed hybrid framework, the ELM module does not directly replace the entire decoder structure of the 3D U-Net architecture. Instead, the trained encoder pathway provides discriminative bottleneck-level latent representations, which are subsequently evaluated using the ELM module for voxel-level analytical classification in the latent feature space. Therefore, the proposed approach should not be interpreted as a direct full-resolution anatomical reconstruction framework based solely on ELM. Rather, the ELM component functions as an analytical decision mechanism operating on compressed latent volumetric representations learned through the encoder–decoder training process. Since these bottleneck representations preserve contextual and spatial information extracted from volumetric CT data, the resulting segmentation outputs still maintain volumetric anatomical consistency while providing improved computational efficiency during the analytical classification stage.

#### 2.2.4. Bottleneck Feature Representation

The bottleneck layer, located at the deepest level of the encoder architecture, contains the most distinctive feature representations learned by the model. This layer forms a dense feature space representing anatomical structures and texture features in the input image. The feature maps in the bottleneck layer can be expressed as in Equation (8):

(8) $$F_{b}\in R^{(C\;\times {\;H}_{b}\;\times {\;W}_{b\;}\times \;D_{b})}$$

Here, C represents the number of channels, and H_b_, W_b_, and D_b_ represent the volume dimensions in the bottleneck layer. The feature maps obtained in this layer contain the strongest representation information that the model has learned.

#### 2.2.5. Creating Feature Vectors

To enable the use of these obtained features in the classification phase, the proposed approach involves rearranging the bottleneck tensor and converting it into a two-dimensional feature matrix format. During this process, each spatial position in the bottleneck feature map is treated as an independent voxel representation. Consequently, the tensor is transformed into a feature matrix as expressed in Equation (9):

F ∈ R^(H^_b_^× W^_b_^× D^_b_^) × C)^ (9)

In this matrix, each row represents the depth feature vector of a voxel, and the columns represent the channel-based feature descriptors learned by the encoder. Accordingly, the feature vector corresponding to the i-th voxel is expressed as in Equation (10):

f_i_ ∈ R^C^, i = 1, 2, …, (H_b_ W_b_ D_b_) (10)

The voxel-level feature vectors obtained in this way are then fed as input to the ELM model in the next stage for classification.

#### 2.2.6. ELM-Based Classification

In the next stage of the proposed method, the resulting feature vectors are evaluated using the ELM model. The ELM model is a feedforward neural network with a single hidden layer and has a very fast training process [30,31]. In the ELM model, the hidden layer outputs are calculated as shown in Equation (11):

H = g(FW + b)(11)

Here, F represents the input feature matrix, W is the random weight matrix between the input and hidden layers, b is the bias term, and g(.) is the activation function. In the ELM model, the output layer weights are calculated using the Moore–Penrose pseudoinverse method as given in Equation (12):

β = H†Y(12)

Here, H† represents the pseudoinverse of the hidden layer output matrix, Y denotes the target label matrix corresponding to voxel-level liver segmentation labels, and β represents the output layer weights. In the experimental configuration, the ELM module was implemented as a single-hidden-layer analytical learning structure consisting of 2048 hidden neurons with sigmoid activation functions. The hidden layer weights were randomly initialized and kept fixed during training, while the output weights were analytically computed using the Moore–Penrose pseudoinverse solution with ridge regularization (λ = 10^−3^).

In this designed structure, the ELM stage acts as a voxel-wise classifier in the latent bottleneck space. Each bottleneck voxel is represented by a channel-wise deep feature vector and evaluated separately within the learned latent feature space during the classification process. Since these representations are generated by the encoder pathway of the 3D U-Net architecture, the feature vectors still preserve contextual and spatial information learned from the volumetric input data.

Although the process performed in the ELM phase is defined as voxel-level classification, this structure should not be considered independent-pixel classification in the classical sense. This is because each voxel is represented by deep feature vectors learned by the 3D U-Net encoder, which contain extensive spatial context. Therefore, the classification process is performed not in the raw image space, but on a latent representation space where spatial relationships and contextual information are embedded. This allows the model to indirectly consider spatial consistency. In this context, the ELM module is positioned not only as a fast classifier, but also as an alternative decision mechanism that enables the analytical evaluation of the learned deep representations. This approach aims to preserve the model’s overall representational power while offering lower computational costs than classical backpropagation-based end-layer optimization. However, it should also be considered that this structure may have limitations, especially in explicitly modeling spatial continuity.

#### 2.2.7. Creating the Segmentation Mask

In the proposed approach, each spatial position in the feature map obtained in the bottleneck layer is treated as an independent feature vector at the voxel level and subjected to separate classification by the ELM module. The predictions obtained as a result of this process are rearranged to be compatible with the volumetric structure in the bottleneck space. Since the bottleneck representation is generated in a compressed latent space, the resulting output corresponds more to a coarse segmentation map rather than a full-resolution mask. Therefore, the proposed framework should be interpreted as a computationally efficient latent-space segmentation strategy rather than a classical full-resolution decoder reconstruction approach. In the final stage, a threshold value of 0.5 is applied to obtain a binary liver segmentation mask. The final segmentation mask was obtained using the thresholding operation as in Equation (13) below:

S(x) = {1, if P(x) ≥ 0.5(13) 0, otherwise}

Here, P(x) represents the probability value predicted by the model, and S(x) represents the generated segmentation mask. As a result of this process, the final segmentation mask representing the liver region is obtained.

## 3. Results

In the experimental studies, both visual and quantitative results are presented to comprehensively evaluate the performance of the proposed method on three-dimensional liver segmentation. In this context, the proposed 3D U-Net–ELM approach was compared with the nnU-Net, 3D U-Net, SwinUNet, and SwinUNet–ELM models. The nnU-Net architecture is known as a self-configuring segmentation framework that automatically adapts preprocessing and network configurations according to dataset characteristics. In this study, the standard configuration provided by the official nnU-Net framework was used to ensure fair experimental comparison. Comparisons considered not only overlap-based metrics but also boundary-based and clinically significant evaluation criteria. Thus, both the volumetric accuracy and the performance in anatomical boundary regions of the models were analyzed in detail.

To ensure a level playing field between models, all methods used the same data preprocessing steps, the same training protocol, and the same basic hyperparameter configurations. All experiments used the Task03_Liver_rs dataset, and performance evaluations were conducted using a fivefold cross-validation approach. This prevented models from showing performance solely based on a specific data split, and also evaluated generalizability and stability across different fold structures. Furthermore, no additional manual editing or post-processing was applied to the test images.

In all models, data loading operations (DataLoader), density clipping ([−1000, 1000] HU), normalization, resizing (resize_factor ≈ 0.5), batch size (1), number of epochs (50), DiceBCELoss loss function consisting of the combination of Dice and BCE losses, Adam optimization algorithm (lr = 5 × 10^−5^), and ReduceLROnPlateau learning rate planner were applied under the same experimental infrastructure. Thus, all models were evaluated under equal experimental conditions. In addition, an early stopping mechanism with a patience value of 10 epochs was consistently applied to all models in order to reduce unnecessary training continuation and limit potential overfitting effects under identical experimental settings. Throughout the experiments, training, validation, and testing performances were monitored together to analyze the model’s convergence behavior and possible overfitting tendencies. The results obtained showed that the model performance generally exhibited a stable structure under different fold structures.

Furthermore, the proposed method was not only based on overlap-based metrics; in addition to basic segmentation metrics such as Dice (DSC), IoU, sensitivity, and F1-score, boundary-based and clinically significant metrics such as HD95, ASD, surface Dice, and volumetric error (%) were also analyzed. Case-based Wilcoxon signed-rank tests were used to assess statistical significance between models. All experiments were performed in the same hardware environment with an Intel i7-14700F processor, 32 GB RAM, and an RTX 4070 Super graphics card. This aimed to reliably compare the models in terms of both segmentation performance and computational efficiency.

### 3.1. Visual Segmentation Results

**Figure 2:**
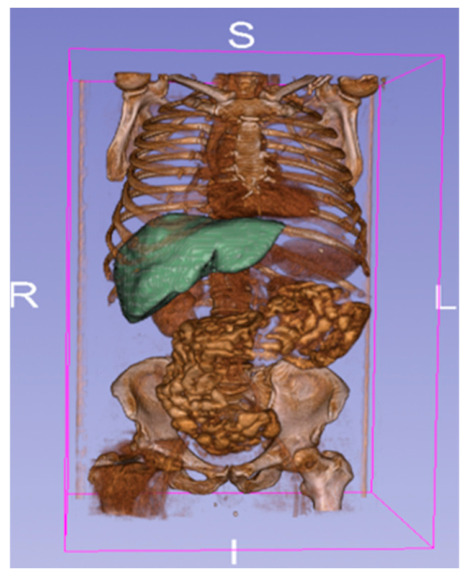
3D abdominal CT + liver label.

The visual results obtained from the experimental studies conducted in this section are presented. Figure 2 is an abdominal CT reconstruction showing the entire skeletal structure and abdominal organs in volumetric 3D form. In addition to this image, the ground truth (label) segmentation of the liver is shown in green. The liver mask shown in the figure was obtained from the label (in nii format) true label images corresponding to the original abdominal CT images in the dataset used during the testing phase.

**Figure 3:**
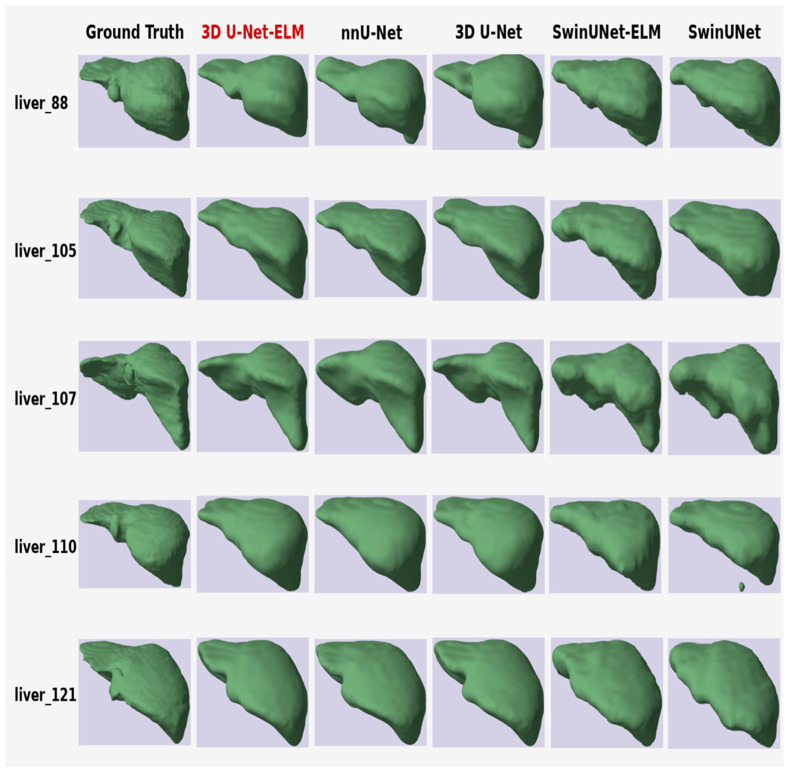
Visual comparison of three-dimensional segmentation results based on front profile for different liver samples.

**Figure 4:**
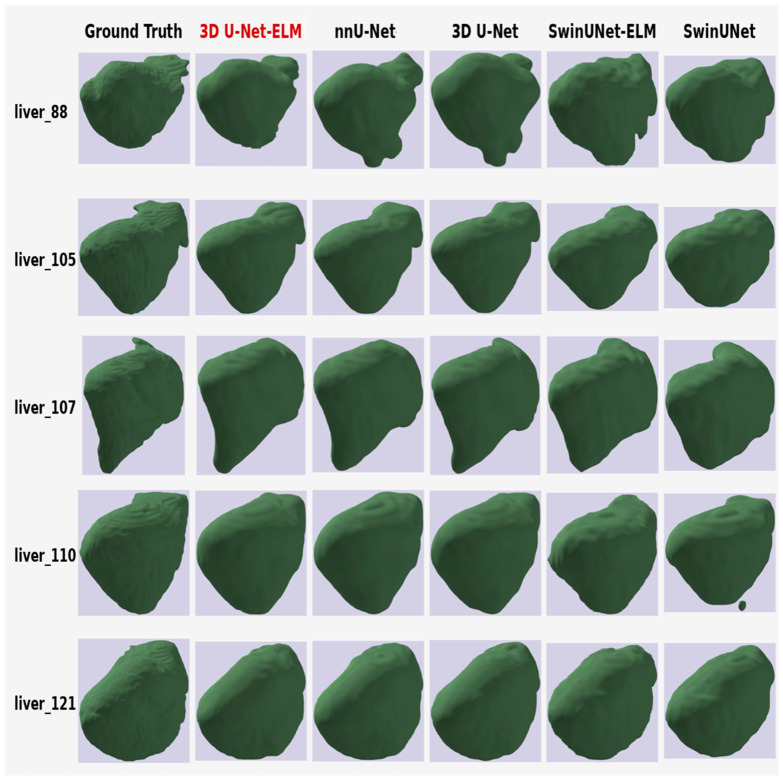
Comparison of three-dimensional liver segmentation results based on side profile view.

**Figure 5:**
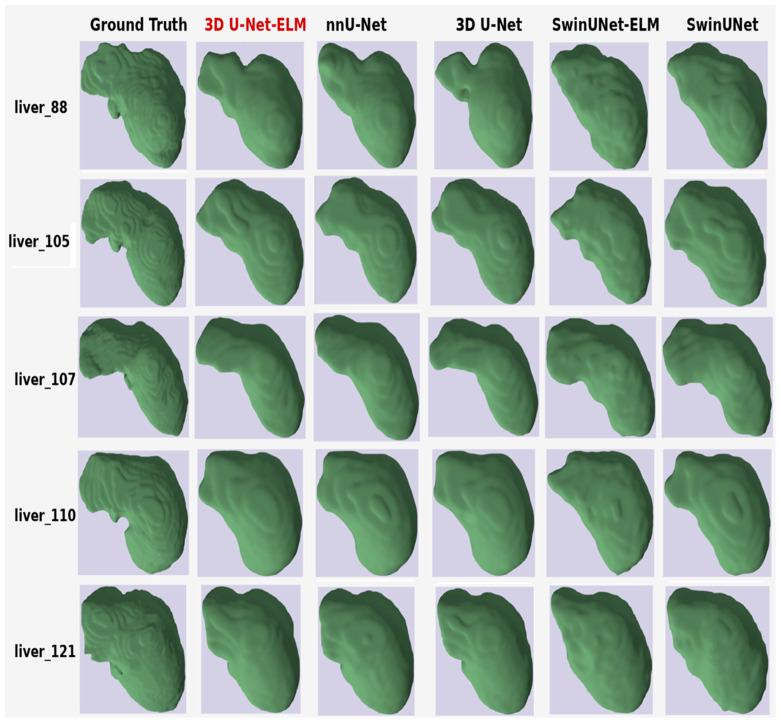
Visual analysis of top profile-based three-dimensional liver segmentation results.

In this study, the three-dimensional segmentation performance of the proposed 3D U-Net–ELM approach on different liver samples was visually evaluated. In this context, the proposed method was compared with the nnU-Net, classical 3D U-Net, SwinUNet–ELM, and SwinUNet models. In the visual analysis, particular attention was paid to the preservation of anatomical integrity, liver surface continuity, preservation of volumetric structures, the success of modeling fine anatomical extensions, and the level of surface irregularities. Figure 3, Figure 4 and Figure 5 present the segmentation results for liver_88, liver_105, liver_107, liver_110, and liver_121 samples from different perspectives.

The Figure 3 presents the segmentation results for the liver_88, liver_105, liver_107, liver_110, and liver_121 samples. The first column shows the segmentation results for the ground truth labels, while the other columns show the outputs for the proposed 3D U-Net–ELM, nnU-Net, 3D U-Net, SwinUNet–ELM, and SwinUNet models, respectively. Examination of the visual results revealed that the proposed 3D U-Net–ELM approach successfully preserves the overall anatomical form and volumetric integrity of the liver. In particular, the fine anatomical extensions and surface transitions in the lower regions of the liver are modeled more balanced and smoother in the proposed method. Furthermore, it is noteworthy that the proposed method maintains surface continuity more stably and limits local surface distortions.

Although the nnU-Net model generally produces strong segmentation results, it can create local volumetric protrusions and surface irregularities in some samples. In the classic 3D U-Net model, anatomical detail losses and softer but volumetrically simplified segmentation structures were observed in certain regions. In some samples, surface deformations, volumetric irregularities, and deviations from anatomical structure became more pronounced in the SwinUNet and SwinUNet–ELM based models. The fact that the proposed hybrid approach produces more balanced segmentation outputs in different liver samples demonstrates that the volumetric representation features learned by the encoder can be effectively evaluated with the ELM-based analytical decision mechanism. These visual results reveal a structure consistent with the overlap-based and boundary-based performance metrics presented in the article.

Figure 4 presents a visual comparison of three-dimensional segmentation results based on side profile views for different liver samples. The figure shows the segmentation results for the liver_88, liver_105, liver_107, liver_110, and liver_121 samples. The first column shows the segmentations corresponding to the ground truth labels. The other columns show the segmentation results produced by the proposed 3D U-Net–ELM, nnU-Net, classic 3D U-Net, SwinUNet–ELM, and SwinUNet models, respectively.

Visual evaluations performed in the side profile view provide important information, particularly regarding the preservation of the volumetric anatomical form of the liver, surface continuity, and the success of modeling fine structural extensions. Examination of the images revealed that the proposed 3D U-Net–ELM approach generally produces more balanced and stable segmentation outputs in different liver samples. It is noteworthy that in the liver_107 and liver_110 samples, the lower appendages and surface transitions of the liver were preserved more closely to the true label segmentations. Although the nnU-Net model generally shows strong segmentation performance, local volumetric protrusions and surface irregularities can occur in some samples. While the classic 3D U-Net model produces smoother surface structures, it can cause volumetric simplification in some anatomical detail regions. In SwinUNet and SwinUNet–ELM based models, more pronounced surface distortions, deviations from anatomical form, and local deformations were observed in certain samples. In particular, in samples liver_88 and liver_110, surface irregularities that may affect volumetric continuity became more pronounced in Swin-based structures. Figure 5 presents a visual comparison of the top profile-based three-dimensional segmentation results for different liver samples. The first column shows the segmentation results for the actual labels (Ground Truth), while the other columns show the outputs for the proposed 3D U-Net–ELM, nnU-Net, 3D U-Net, SwinUNet–ELM, and SwinUNet models, respectively.

Evaluations based on the top view show that the proposed 3D U-Net–ELM approach preserves the overall anatomical form, surface continuity, and volumetric integrity of the liver in a more balanced way. It is particularly noteworthy that in the liver_105, liver_110, and liver_121 samples, the proposed method creates surface structures closer to Ground Truth segmentations. In contrast, some comparison models showed local surface irregularities, volumetric distortions, and deviations from anatomical structure.

Table 2 presents the overlap-based performance results obtained from fivefold cross-validation experiments of the nnU-Net, 3D U-Net, SwinUNet, and SwinUNet-ELM models, used for comparison with the proposed 3D U-Net–ELM model. Dice (DSC), IoU, sensitivity, and F1-score metrics were used to evaluate segmentation performance. Examination of the results showed that the proposed 3D U-Net–ELM approach produced the highest results in all key performance metrics. In particular, the low standard deviation values indicate that the model can produce more stable and generalizable results across different fold structures. Furthermore, it was observed that although the nnU-Net model had a strong baseline, the proposed hybrid approach achieved higher overlap performance. The Transformer-based SwinUNet model, on the other hand, exhibited a more unstable structure, particularly due to its low Dice and IoU values.

To quantify the practical significance of performance differences, Cohen’s d effect sizes were computed between the proposed 3D U-Net–ELM model and all comparison models. The statistical analysis results are presented in Table 3. Effect size analysis is particularly appropriate when per-fold raw prediction distributions are unavailable for formal non-parametric statistical testing, as it provides a sample-size-independent measure of the magnitude of observed differences.

Cohen’s d was calculated using the pooled standard deviation of each model pair (Equations (14) and (15)).

d = (μ_1_ − μ_2_)/σ_pooled (14)

σ_pooled = √[(σ_1_^2^ + σ_2_^2^)/2] (15)

Positive mean and positive d values indicate superior performance of the proposed model. For metrics where lower values indicate better performance (HD95, ASD, and volumetric error), the sign of d was reversed accordingly. Effect size magnitudes were categorized as small, medium, and large according to commonly used thresholds.

The proposed 3D U-Net–ELM model demonstrated large to very large effect sizes against 3D U-Net (d = 1.52 for Dice, d = 1.69 for IoU), SwinUNet-ELM (d = 2.30 and 2.84), and SwinUNet (d = 3.21 and 3.62), indicating practically meaningful superiority beyond numerical differences. Against nnU-Net, a large effect was observed for Dice and IoU (d = 0.83 and 0.91, respectively), while a small effect was noted for sensitivity (d = 0.39), confirming that the performance gap was smallest against this strong baseline.

Table 4 presents the results obtained not only through overlap-based performance but also through boundary-based and clinically significant metrics. The HD95 and ASD metrics evaluate the distance of segmentation boundaries from actual anatomical boundaries, while the surface Dice metric measures the concordance of segmentation surfaces. The volumetric error (%) metric shows the difference between the predicted liver volume and the actual volume. When the results are examined, it can be seen that the proposed 3D U-Net–ELM approach produces lower HD95 and ASD values while achieving higher surface Dice scores. Furthermore, the lower volumetric error rate indicates that the proposed hybrid structure can predict liver volume more accurately and consistently. Although the nnU-Net model showed strong boundary performance, the proposed model achieved more successful results in all boundary-based metrics.

### 3.2. Computational Efficiency and Complexity Analysis

Table 5 compares the approximate single-run training times of the nnU-Net, 3D U-Net, SwinUNet, and SwinUNet-ELM models used for comparison with the proposed 3D U-Net–ELM model. All models were evaluated in the same hardware environment and under similar training configurations. The results show that the proposed hybrid approach requires a shorter training time, especially compared to Transformer-based models. The nnU-Net model, used as a robust CNN-based baseline, was observed to perform more efficiently than the classical 3D U-Net model. Furthermore, the proposed 3D U-Net–ELM approach achieved higher segmentation performance with a shorter training time compared to the nnU-Net model. The SwinUNet model, in particular, requires a significantly longer training time than the proposed method. These results demonstrate that the ELM-based analytical learning mechanism can provide a more efficient learning process by reducing the computational costs.

Table 6 details the computational complexity and hardware requirements of the proposed 3D U-Net–ELM model. The model contains approximately 5.67 million trainable parameters and has a computational complexity of approximately 236 GFLOPs. It can also operate with an inference time of approximately 0.079 s per case and approximately 1440 MB of GPU memory usage. The fact that the ELM-based learning phase takes only about 14 s shows that the hybrid structure imposes a limited overhead on the total training time. These results demonstrate that the proposed method offers a balanced structure in terms of both segmentation performance and computational efficiency.

An additional ablation study was conducted to evaluate the effect of intensity preprocessing steps on segmentation performance in the proposed 3D U-Net–ELM architecture. The results are presented in Table 7. In this study, HU clipping and intensity normalization operations were removed, but the spatial resizing operation was retained to maintain the same input resolution and computational cost. Thus, the contribution of preprocessing was analyzed in a more controlled manner. The results show that intensity preprocessing operations significantly contribute to the performance, especially on boundary-based metrics. When preprocessing was removed, an increase in HD95 and ASD values and a decrease in surface Dice score were observed. However, the proposed 3D U-Net–ELM structure performed better than the 3D U-Net approach with and without preprocessing.

## 4. Discussion

Artificial intelligence-based methods have been frequently used in recent years, especially in the biomedical field. In this study, an automated artificial intelligence approach was used for liver segmentation [32,33]. Liver segmentation is a critical assessment tool that provides the quantitative basis for surgical decision-making. In hepatectomy planning, calculations of total liver volume and future liver remnant (FLR) are crucial for assessing resectability and predicting the risk of post-hepatectomy liver failure [2,34]. Even small errors in segmentation can lead to clinically significant deviations in volumetric analyses, affecting surgical strategy and operability limits [1,34].

The necessity of liver segmentation is even more evident in liver transplantation practice. Especially in living donor evaluations, the accurate calculation of graft volume and remaining liver reserve in the donor is critical for calculating sufficient liver volume for the recipient and for donor safety [35,36]. Segmentation quality is a decisive parameter in reliably establishing a volume-based risk balance [3,37].

In hepatocellular carcinoma cases, monitoring treatment response and targeted downstaging after locoregional treatments is closely linked to the accuracy of segmentation-based measurements [1,36]. Segmentation-related errors can lead to the misinterpretation of treatment response and clinical decision uncertainty by causing deviations in tumor size and tumor burden assessment [38]. The clinical impact of segmentation errors is particularly pronounced in anatomical regions with low margin contrast. Systematic deviations in contact surfaces with adjacent organs can directly affect volumetric analyses and tumor burden assessment. Therefore, an increase in margin sensitivity is clinically significant. However, external validation across different imaging protocols, devices, and patient subgroups is necessary to strengthen the model’s clinical validity [39]. In addition, it is important to validate segmentation performance with clinical outcome measures [40].

In this study, model performance was evaluated using a fivefold cross-validation protocol in order to reduce split dependency and assess the robustness and generalization capability of the proposed approach under different fold configurations. The experimental results demonstrated that the proposed method produced stable and competitive segmentation performance across different folds. Nevertheless, further validation studies on larger multicenter datasets and different imaging protocols are still required to comprehensively evaluate the clinical generalizability of the model.

To ensure a fairer and more transparent comparison with studies in the literature, Table 8 was prepared considering not only Dice scores but also the dataset structures, image resolutions, segmentation targets, and evaluation protocols used. While some of the studies examined were performed on the original high-resolution MSD Task03_Liver dataset, this study used the scaled-down and lower-resolution Task03_Liver_rs dataset to reduce computational cost and memory usage. Some of the methods in the literature focus on liver organ segmentation, while others address liver + tumor or liver lesion segmentation together. However, all relevant studies were developed within the scope of volumetric segmentation problems related to the liver region, and liver segmentation performance was reported separately in most studies. Therefore, the methods were included in the table to evaluate the general volumetric segmentation performance trends involving the liver region. In addition, the evaluation protocols used also differed among the studies. While some methods preferred fixed test-reference, some studies used few-shot learning scenarios or cross-validation-based evaluation protocols. Therefore, when making direct numerical comparisons between different studies, it should be considered that experimental conditions and dataset configurations may differ. Nevertheless, the results obtained by the proposed 3D U-Net–ELM approach in fivefold cross-validation experiments show that the method offers a competitive structure in terms of both segmentation performance and computational efficiency.

Differences in preprocessing pipelines, image resolutions, and label handling strategies across studies may limit direct one-to-one comparison with previously reported results in the literature. Therefore, literature-based comparisons in this study should be interpreted as general performance references rather than absolute benchmarking results. Nevertheless, all models evaluated within this study were trained and tested under identical experimental conditions to ensure a fair internal comparison.

## 5. Conclusions

In this study, a robust hybrid approach combining the powerful feature extraction capability of the 3D U-Net architecture with the rapid analytical learning ability of the ELM approach is proposed for liver segmentation in 3D CT images. Experimental studies conducted on the Task03_Liver_rs dataset using a fivefold cross-validation strategy demonstrated that the proposed 3D U-Net–ELM framework achieved competitive and stable segmentation performance. According to the obtained results, the proposed model achieved a mean Dice score of 0.9399 ± 0.0210 and outperformed standard 3D U-Net, SwinUNet, and SwinUNet–ELM architectures in both overlap-based and boundary-based evaluation metrics. In addition to the Dice and IoU metrics, lower HD95 and ASD values together with higher surface Dice scores indicate that the proposed framework provides more consistent boundary delineation performance. These findings suggest that combining deep learning-based volumetric representation learning with analytical machine learning approaches can provide an effective balance between segmentation performance and computational efficiency. The results also indicate that ELM integration contributes to more efficient use of computational resources while maintaining competitive segmentation accuracy. Furthermore, the proposed hybrid structure demonstrated stable segmentation performance even in challenging low-contrast regions and anatomically complex liver boundaries. Overall, the proposed framework offers a computationally efficient alternative to conventional deep learning-based volumetric segmentation approaches by combining the representational capability of convolutional neural networks with the fast analytical learning structure of ELM. Although the proposed method produced stable results under fivefold cross-validation experiments, the absence of independent multicenter external validation remains an important limitation of this study. Future studies will focus on evaluating the proposed framework on larger and more diverse multicenter datasets obtained from different clinical environments and imaging protocols. In addition, future work may investigate more advanced optimization strategies for ELM parameter selection and the applicability of the proposed framework to different organ segmentation problems.

## Figures and Tables

**Table 1:** Comparison of the original MSD Task03_Liver and the Task03_Liver_rs datasets used.

Feature	Original MSD Task03_Liver Data Set	Data Set Used (Task03_Liver_rs)
Number of labeled volumes	131	123 (8 low-quality cases were excluded)
Test volume	70 (unlabeled)	Not used
File size	~27–30 GB	9.44 GB
Resolution	Full (full-resolution)	Approximately half resolution (resize_factor ≈ 0.5–0.6)
Pre-processing	None	Resize + density normalization + cleaning

**Table 2:** Overlap-based performance metrics.

Model	Dice (DSC)	IoU	Sensitivity
3D U-Net-ELM	0.9399 ± 0.0210	0.8874 ± 0.0358	0.9416 ± 0.0260
nnU-Net	0.9172 ± 0.0325	0.8486 ± 0.0487	0.9281 ± 0.0413
3D U-Net	0.9018 ± 0.0285	0.8220 ± 0.0415	0.9194 ± 0.0317
SwinUNet-ELM	0.8673 ± 0.0394	0.7655 ± 0.0489	0.8540 ± 0.0445
SwinUNet	0.8126 ± 0.0521	0.6914 ± 0.0678	0.7945 ± 0.0582

**Table 3:** Cohen’s d effect sizes—Overlap-based metrics (Dice, IoU, sensitivity, F1-score).

Comparison (vs. 3D U-Net-ELM)	Metric	Mean	Cohen’s d	Effect Size
nnU-Net	Dice	+0.0227	0.830	Large
	IoU	+0.0388	0.908	Large
	Sensitivity	+0.0135	0.391	Small
	F1-Score	+0.0227	0.830	Large
3D U-Net	Dice	+0.0381	1.522	Very Large
	IoU	+0.0654	1.688	Very Large
	Sensitivity	+0.0222	0.766	Medium
	F1-Score	+0.0381	1.522	Very Large
SwinUNet-ELM	Dice	+0.0726	2.300	Very Large
	IoU	+0.1219	2.845	Very Large
	Sensitivity	+0.0876	2.404	Very Large
	F1-Score	+0.0726	2.300	Very Large
SwinUNet	Dice	+0.1273	3.205	Very Large
	IoU	+0.1960	3.615	Very Large
	Sensitivity	+0.1471	3.264	Very Large
	F1-Score	+0.1273	3.205	Very Large

**Table 4:** Boundary-based and clinical metrics.

Model	HD95 (mm)	ASD (mm)	Surface Dice	Vol. Error (%)
3D U-Net-ELM	3.66 ± 1.18	1.08 ± 0.35	0.9483 ± 0.0205	6.81 ± 2.69
nnU-Net	4.08 ± 1.92	1.24 ± 0.42	0.9317 ± 0.0348	10.92 ± 5.84
3D U-Net	5.28 ± 1.62	1.67 ± 0.46	0.9085 ± 0.0308	10.25 ± 3.62
SwinUNet-ELM	6.84 ± 2.08	2.04 ± 0.63	0.8812 ± 0.0406	13.05 ± 4.41
SwinUNet	9.12 ± 3.01	3.04 ± 0.95	0.8246 ± 0.0542	17.85 ± 6.30

**Table 5:** Comparison of training duration and computational efficiency.

Method	Approximate Single-Run Training Duration (min)	Total Duration (Hour:Minute)	Difference According to Proposed Model	Relative Slowdown
Proposed 3D U-Net + ELM (Reference)	29.17 min	0:29	0 min	0%
nnU-Net	33.42 min	0:33	+4.25 min	14.6% slower
3D U-Net	36.67 min	0:36	+7.5 min	25.7% slower
SwinUNet-ELM	45 min	0:45	+15.83 min	54.3% slower
SwinUNet	65.83 min	1:05	+36.66 min	125.6% slower

**Table 6:** Computational complexity analysis of the proposed 3D U-Net–ELM model.

Metric	Proposed 3D U-Net–ELM
Trainable Parameters	5,667,169
GFLOPs	236.16
Inference Time/Case	0.0793 s
GPU Peak Memory Usage	1440.5 MB
Approximate Single-Run Training Duration	29.17 min
ELM Training Time	14.36 s
Hidden Neurons (ELM)	2048
Input Resolution	128 × 128 × 64
Batch Size	1

**Table 7:** Ablation analysis of intensity preprocessing and ELM contribution in the proposed 3D U-Net–ELM framework.

Configuration	Preprocessing	ELM	Dice (DSC)	IoU	HD95 (mm)	ASD (mm)	Surface Dice	Vol. Error (%)
3D U-Net	Yes	No	0.9018 ± 0.0285	0.8220 ± 0.0415	5.28 ± 1.62	1.67 ± 0.46	0.9085 ± 0.0308	10.25 ± 3.62
3D U-Net–ELM without intensity preprocessing	No	Yes	0.9050 ± 0.0265	0.8290 ± 0.0398	5.84 ± 1.74	1.82 ± 0.49	0.9015 ± 0.0287	9.74 ± 3.12
Proposed 3D U-Net–ELM	Yes	Yes	0.9399 ± 0.0210	0.8874 ± 0.0358	3.66 ± 1.18	1.08 ± 0.35	0.9483 ± 0.0205	6.81 ± 2.69

**Table 8:** Comparison of the proposed method with recent liver segmentation studies reported in the literature in terms of Dice (DSC) performance.

Year	Model	Dataset/Resolution	Task	Evaluation Protocol	Dice (DSC) (%)
2026	HMC-Transducer [41]	MSD-Liver + LiTS	Liver + tumor	Cross-validation	89.67 ± 1.25
2025	STD-Net [42]	MSD Task03 Liver	Liver lesion	Test split	91.9
2023	TransU2-Net [43]	Original high-resolution MSD Task03_Liver (512 × 512)	Liver organ	Test split	96.24
2025	FSS-ULivR [40]	Original high-resolution MSD Task03_Liver (512 × 512)	Liver organ	Few-shot evaluation	94.05 ± 0.73
2026	Proposed 3D U-Net–ELM	Downsampled MSD Task03_Liver_rs	Liver segmentation	Fivefold cross-validation	0.9399 ± 0.0210

## Data Availability

The data presented in this study are openly available [Kaggle] [https://www.kaggle.com/datasets/prathamgrover/3d-liver-segmentation, accessed on 15 January 2026 [19]].
